# The Diversity and Metabolism of Culturable Nitrate-Reducing Bacteria from the Photic Zone of the Western North Pacific Ocean

**DOI:** 10.1007/s00248-023-02284-w

**Published:** 2023-08-08

**Authors:** Zhichen Jiang, Sizhen Liu, Dechao Zhang, Zhongli Sha

**Affiliations:** 1 Laboratory of Marine Organism Taxonomy and Phylogeny, Qingdao Key Laboratory of Marine Biodiversity and Conservation, Institute of Oceanology, Chinese Academy of Sciences, Qingdao, 266071 China; 2 Laoshan Laboratory, Qingdao, 266237 China; 3 University of Chinese Academy of Sciences, Beijing, 100049 China; 4 National Key Laboratory of Agricultural Microbiology, Huazhong Agricultural University, Wuhan, 430070 China; 5 Hubei Key Laboratory of Agricultural Bioinformatics, College of Informatics, Huazhong Agricultural University, Wuhan, 430070 China

**Keywords:** Diversity, Nitrate-reducing bacteria, The photic zone, Pacific Ocean

## Abstract

To better understand bacterial communities and metabolism under nitrogen deficiency, 154 seawater samples were obtained from 5 to 200 m at 22 stations in the photic zone of the Western North Pacific Ocean. Total 634 nitrate-utilizing bacteria were isolated using selective media and culture-dependent methods, and 295 of them were positive for nitrate reduction. These nitrate-reducing bacteria belonged to 19 genera and 29 species and among them, *Qipengyuania flava*, *Roseibium aggregatum*, *Erythrobacter aureus*, *Vibrio campbellii*, and *Stappia indica* were identified from all tested seawater layers of the photic zone and at almost all stations. Twenty-nine nitrate-reducing strains representing different species were selected for further the study of nitrogen, sulfur, and carbon metabolism. All 29 nitrate-reducing isolates contained genes encoding dissimilatory nitrate reduction or assimilatory nitrate reduction. Six nitrate-reducing isolates can oxidize thiosulfate based on genomic analysis and activity testing, indicating that nitrate-reducing thiosulfate-oxidizing bacteria exist in the photic zone. Five nitrate-reducing isolates obtained near the chlorophyll *a*-maximum layer contained a dimethylsulfoniopropionate synthesis gene and three of them contained both dimethylsulfoniopropionate synthesis and cleavage genes. This suggests that nitrate-reducing isolates may participate in dimethylsulfoniopropionate synthesis and catabolism in photic seawater. The presence of multiple genes for chitin degradation and extracellular peptidases may indicate that almost all nitrate-reducing isolates (28/29) can use chitin and proteinaceous compounds as important sources of carbon and nitrogen. Collectively, these results reveal culturable nitrate-reducing bacterial diversity and have implications for understanding the role of such strains in the ecology and biogeochemical cycles of nitrogen, sulfur, and carbon in the oligotrophic marine photic zone.

**Supplementary Information:**

The online version contains supplementary material available at 10.1007/s00248-023-02284-w.

## Introduction

On Earth, the nitrogen (N) cycle has evolved over ~2.7 billion years through biogeochemical and microbial processes that are coupled via robust natural feedbacks and controls [1]. N is often a primary limiting nutrient for marine microbial growth/metabolism [2]. In coastal and upwelling areas, the level of dissolved inorganic N (DIN), such as NO_3_^−^ or NO_2_^−^, is usually sufficient to support microbial growth; most tropical and subtropical oceans, however, are oligotrophic with undetectable levels of DIN [2, 3]. In the oligotrophic tropical ocean, nitrate mainly arises from euphotic zone nitrification and the deep ocean [4]. The relative flow of NO_3_^−^ which can be used as nitrogen source for growth through assimilation and reduction by bacteria largely determines the composition of the upper ocean’s N pool [5]. Bacteria are more prevalent in the photic zone (upper ca. 200 m) of the ocean than in deep waters, and the total number of bacteria decreases with depth [6]. N cycle is driven by complex microbial biogeochemical transformations and among them, nitrate-reducing bacteria constitute ca. 50% of microbial population present in aquatic environment [7]. Nitrate reduction is involved in both assimilatory nitrate reduction (ANR) and dissimilatory nitrate reduction. ANR converts nitrate to ammonium (via nitrite) which can be subsequently incorporated into cell biomass, while dissimilatory nitrate reduction includes denitrification, the respiration of nitrate to nitrogen gas, and dissimilatory nitrate reduction to ammonia (DNRA) [8]. There are three distinct types of nitrate reductases in prokaryotes, including periplasmic (Nap), membrane-bound (Nar), and assimilatory (Nas) nitrate reductase [9]. Many studies have demonstrated the diversity of the nitrate reductase genes [10–15] and nitrate-reducing community [16–18] by using functional molecular markers (e.g., *napA*, *narG*, *narB*, and *nasA*). Previous research has demonstrated that denitrification typically prevails in the reduction of nitrate when the concentration of nitrate is high and the availability of organic carbon is limited [19]. Conversely, DNRA becomes the dominant nitrate reduction process when the concentration of nitrate is low and organic carbon is abundant [19]. The cycling of sulfur is also crucial in waters, as it facilitates the interplay between the generation and consumption of hydrogen sulfide (H_2_S) [20]. The nitrogen and sulfur cycles interconnect through the competition for easily degradable forms of organic carbon between nitrate-reducing and sulfate-reducing bacteria [21]. Although surveys of the diversity of culturable nitrate-reducing bacteria have been carried out in oxygen-deficient marine systems [22–25], there is relatively limited research on the diversity of culturable nitrate-reducing bacteria under N-deficient conditions and their role in nitrogen, sulfur, and carbon metabolism.

The Western North Pacific Ocean (WNPO) is one of the world’s largest oligotrophic regions; it is characterized by low primary production, and thus is regarded as an ideal region for studying the metabolism of microorganisms under N-deficient conditions [2, 26]. Previous studies on prokaryotes of the water column in the North Pacific Ocean were conducted using culture-independent methods, such as ribosomal tag pyrosequencing, real-time qPCR, and metaproteomic analyses [2, 27–31]. One significant disadvantage of molecular techniques that rely on genomic DNA is their inability to differentiate between DNA sourced from non-viable cells, viable cells that are not cultivable, and metabolically viable cells [29]. Admittedly, culture-based methods have some limitations, such as the inability to culture the majority of organisms; or organisms that have mutualisms with others cannot live in isolation. However, when it comes to ecological exploration, culture-based studies can enable researchers to perform physiological tests using culturable microorganisms, and thereby infer the detailed ecological roles of the microorganisms [25]. Studying the diversity of nitrate-reducing bacteria is crucial because nitrogen is frequently a key nutrient that limits microbial growth and metabolism. Meanwhile, nitrate-reducing bacteria also participate in biogeochemical process of the carbon and sulfur cycles, which can contribute to maintain ecosystem stability [32]. Here, we sought to isolate nitrate-reducing bacteria from the photic zone of WNPO by using selective media and nitrate reduction tests, and then apply genomic analyses to elucidate their metabolisms. This study provides insights into the physiological traits of nitrate-reducing bacteria inhabiting these areas, detailing their metabolic role in biogeochemical cycles of nitrogen, sulfur, and carbon.

## Materials and Methods

### Study Areas and Sample Collection

**Fig. 1:**
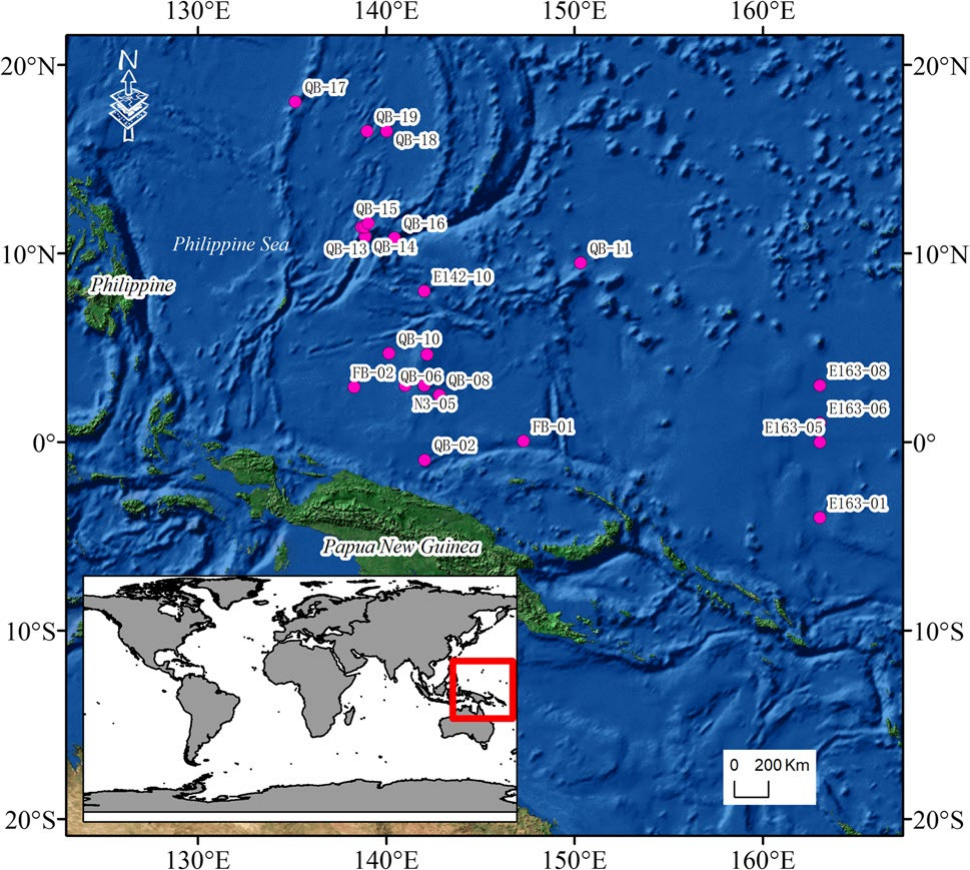
The 22 sampling locations in the Western North Pacific Ocean. The pink dots represent the sampling location. The red rectangle represents the sampling region

From March to May 2021, a 46-day expedition in the WNPO was carried out by the Institute of Oceanology, Chinese Academy of Sciences, on the scientific research ship, *Science* (Fig. 1). A total of 154 seawater samples were collected from 5 to 200 m at 22 stations with Niskin bottles attached to a rosette sampling system equipped with a Sea-Bird SBE911 CTD. The chlorophyll *a* (Chl-*a*) were measured using a Wet Star fluorometer attached to the Sea-Bird SBE911 CTD (Table S1). On board the boat, seawater sample (200 μL) was plated on nitrate agar medium (NAM; 0.2% KNO_3_, 0.02% MgSO_4_·7H_2_O, 0.08% K_2_HPO_4_·3H_2_O, 2% NaKC_4_H_4_O_6_, 1% NaCl, 2% agar). After samples were incubated at 25°C in a constant temperature incubator for 7–14 days, single colonies were selected, cultured in medium (0.1% KNO_3_, 1% peptone, 2% agar, sterile seawater), and stored as a suspension in 20% (w/v) glycerol at −80°C.

### Phylogenetic Analysis of Nitrate-Utilizing Bacteria

For DNA extraction, all isolates were grown for 3–5 days in 5 mL liquid medium (0.1% KNO_3_, 1% peptone, sterile seawater) and incubated at 25 °C. Genomic DNA was extracted using an UltraClean Microbial DNA isolation kit (Mo Bio Laboratories) according to the manufacturer’s protocol. The gene encoding for the 16S rRNA was amplified by PCR with the forward primer 27F AGAGTTTGATCCTGGCTCAG and reverse primer 1541R AAGGAGGTGATCCAGCCGCA [33]. Strains were assigned to species with a cutoff of 98% similarity in 16S rRNA gene sequences [34]. To clarify the taxonomic status and evolutionary relationship among different nitrate-utilizing bacteria, 16S rRNA sequence of different species was aligned by MAFFT v7.4.8 [35]. A phylogenetic tree based on this alignment was constructed using IQ-tree v2.0.3 [36] with 1000 bootstrap replicates, employing the TIM3+F+R5 model; Interactive Tree Of Life (iTOL) was used to visualize the phylogenetic tree [37].

### Screening of Nitrate-Reducing Bacteria

Nitrate-reducing bacteria were screened from nitrate-utilizing strains by checking their ability to reduce nitrate according to previously described procedures [25, 38]. Briefly, nitrate-utilizing strains were transferred to the liquid medium (0.1% KNO_3_, 1% peptone, sterile seawater) and incubated at 25°C for 7 days, and then 2 drops each of Griess A reagent (sulfanilamide with 5 mol/L acetic acid) and Griess B reagent (*N*-(1-naphthyl) ethylenediamine with 5 mol/L acetic acid) were sequentially added to the liquid medium. The negative control tube only contained sterile nitrate liquid medium. If the test sample turned red, pink, or orange, there was nitrite in the tube and the isolate was scored as a nitrate reducer. If no color appeared, zinc dust was added. The lack of any color change at this point was taken as indicating that there was no residual nitrate in the liquid medium, meaning that the strain could completely reduce nitrate. If the liquid medium changed at this point from colorless to red, pink, or orange (as seen for the control), the strain was considered to be unable to reduce nitrate.

### The Ability to Oxidize Thiosulfate for Nitrate-Reducing Bacteria

To validate the potential ability to oxidize thiosulfate, we incubated nitrate-reducing strains in modified DSMZ (Deutsche Sammlung von Mikroorganismen und Zellkulturen GmbH) 142 liquid medium (0.1% (NH_4_)_2_SO_4_, 0.15% MgSO_4_·7H_2_O, 0.42% CaCl_2_.2H_2_O, 0.05% K_2_HPO_4_, 0.1% Na_2_S_2_O_3_.5H_2_O, vitamins, trace elements, sterile seawater) containing phenol red (0.3 mg/L) at 25°C for 3–5 days, and monitored changes in pH values. The reduced sulfur source for autotrophic growth was sodium thiosulfate. When the liquid turned from red to yellow, the change was taken as indicating that the pH value decreased and the strain effectively oxidized thiosulfate.

### Genome Sequencing, Assembly, and Annotation of Nitrate-Reducing Bacteria

Genomic DNA of nitrate-reducing strains representing different species was extracted as described above. A paired-end library with an insert size of 350 bp was constructed for each genome, and sequencing was performed by using Illumina NovaSeq 6000 platform [39]. The raw reads of each genome were processed for removal of low-quality bases and adaptors to obtain the clean reads, using Trimmomatic v0.36 [40]. The resulting 150 bp paired-end reads with about 200× were quality checked and assembled using FastQC (v0.11.9) and SPAdes genome assembler v3.15.2 [41]. The quality of each assembly was evaluated by BUSCO (5.0.0) [42]. Gene prediction and genome annotation were performed using Prokka v1.14.6 [43].

### Nucleotide Sequence Data

The sequence data generated in this study have been deposited to GenBank under the accession numbers OP835944-OP836037 for 16S rRNA gene sequences of 94 nitrate-utilizing strains and under BioProjectID PRJNA882570 for genome sequences of 29 nitrate-reducing strains.

### Functional Annotation of Genes for Different Metabolism Types

Carbohydrate-active enzymes (CAZymes) were annotated using dbCAN (v2.0.11) [44] against the CAZyme database v9. To identify genes encoding proteases, all of the predicted genes were searched against the peptidase database, MEROPS [45], using DIAMOND [46] BLASTP (*E*-value 10^−12^). Genes belonging to different carbohydrate-active enzymes or protease families were classified using in-house python scripts. The python scripts used have been uploaded to the code hosting platform github https://github.com/liusizhenssh/cazy_classification.

Annotation of the predicted proteins was performed using eggNOG-mapper (v2.0.1) [47] with the DIAMOND mapping mode, based on the eggnog 5.0 orthology data. Genes belonging to different types of nitrogen and sulfur metabolism were classified by manual selection according to the results of eggNOG-mapper. To identify dimethylsulfoniopropionate (DMSP) synthesis and cleavage genes, alignments of ratified sequences of all genes of interest [48, 49] were analyzed by BLASTP searches against the RefSeq database with parameters *E*-value ≤10^−12^ and identity ≥70%, and manual annotation was used to verify the top hits.

## Results and Discussion

### Diversity and Distribution of the Culturable Nitrate-Reducing Strains

A total of 728 nitrate-utilizing bacteria were isolated from 154 seawater samples. After removing duplicate bacterial strains from the same water sample using a 100% identity cutoff in 16S rRNA gene sequences, 634 nitrate-utilizing strains belonging to 94 species were obtained (Fig. S1). 295/634 nitrate-utilizing bacteria belonging to 19 genera and 29 species were positive for nitrate reduction. The distribution and abundance of 29 nitrate-reducing strains representing different species with the ability to reduce nitrate in different seawater layers and at different stations are presented in Fig. S2. The nitrate-reducing strains were found in the highest proportion at station E163-01 (17/295), station E163-05 (17/295), and station E163-08 (17/295), while the minimum number of nitrate-reducing strains was observed at station QB-08 (7/295) (Table S2). In terms of vertical distribution, the number of nitrate-reducing strains showed an increasing trend with depth, followed by a decreasing trend. They were most frequently detected in the middle of the photic zone (100 m) (52/295) (Table S3).

Remarkably, *Qipengyuania flava* (formerly called *Erythrobacter flavus*), *Roseibium aggregatum* (formerly called *Stappia aggregate*), *Erythrobacter aureus*, *Vibrio campbellii*, and *Stappia indica* were found in all of the sampled seawater layers and at almost all of the stations where the isolates were obtained. *Q*. *flava* has been isolated from marine environments and a member of this species was reported to produce sulfur-containing carotenoids whose main functions are light harvesting and photoprotection during photosynthesis in the photic zone [50–52]. In this study, *Q*. *flava* was widely distributed at almost all stations (21/22) and in the whole water column (especially 5–50m). *Stappia* isolates can respire nitrate or perform denitrification; this facilitates their participation in nitrogen cycling under aerobic and anaerobic processes in marine environments [53]. *S. indica* recently has been reported as a human pathogen causing peritonitis [54] and was mostly found in the layers deeper than 75m in this study. *R. aggregatum* IAM 12614^T^ could reduce nitrate to gas [55] and was mainly isolated from deeper layers (100–200m) in this study. Variable results have been reported for tests of nitrate reduction among *Erythrobacter* strains [50, 56]. *V. campbellii* is widely distributed in tropical and temperate marine environments; *V. campbellii* BAA-1116 reportedly harbors a functional proteorhodopsin and retinal biosynthesis gene cluster that enables it to exploit light as an energy source in the photic zone [57, 58]. Photosynthetic microorganisms are able to thrive in the euphotic zone, including *Prochlorococcus* [59] or aerobic anoxygenic phototrophic bacteria [60]. Only enzymes associated with the ATP synthase complex were detected in this study, whereas enzymes involved in other photosynthetic pathways were not present, suggesting that nitrate-reducing strains were incapable of performing photosynthesis (Fig. S3). Previous research has reported that specific oceanic bacteria, like *Methylococcales*, had the ability to utilize methane as both an energy and carbon source [61]. These bacteria converted methane into carbon dioxide through methane monooxygenase. We found that all nitrate-reducing strains did not contain methane monooxygenase or other enzymes involved in complete methane conversion to carbon dioxide (Fig. S4). Therefore, these nitrate-reducing strains did not possess the capability for methane metabolism in the marine environment.

### Nitrogen Metabolism of Nitrate-Reducing Strains

**Fig. 2:**
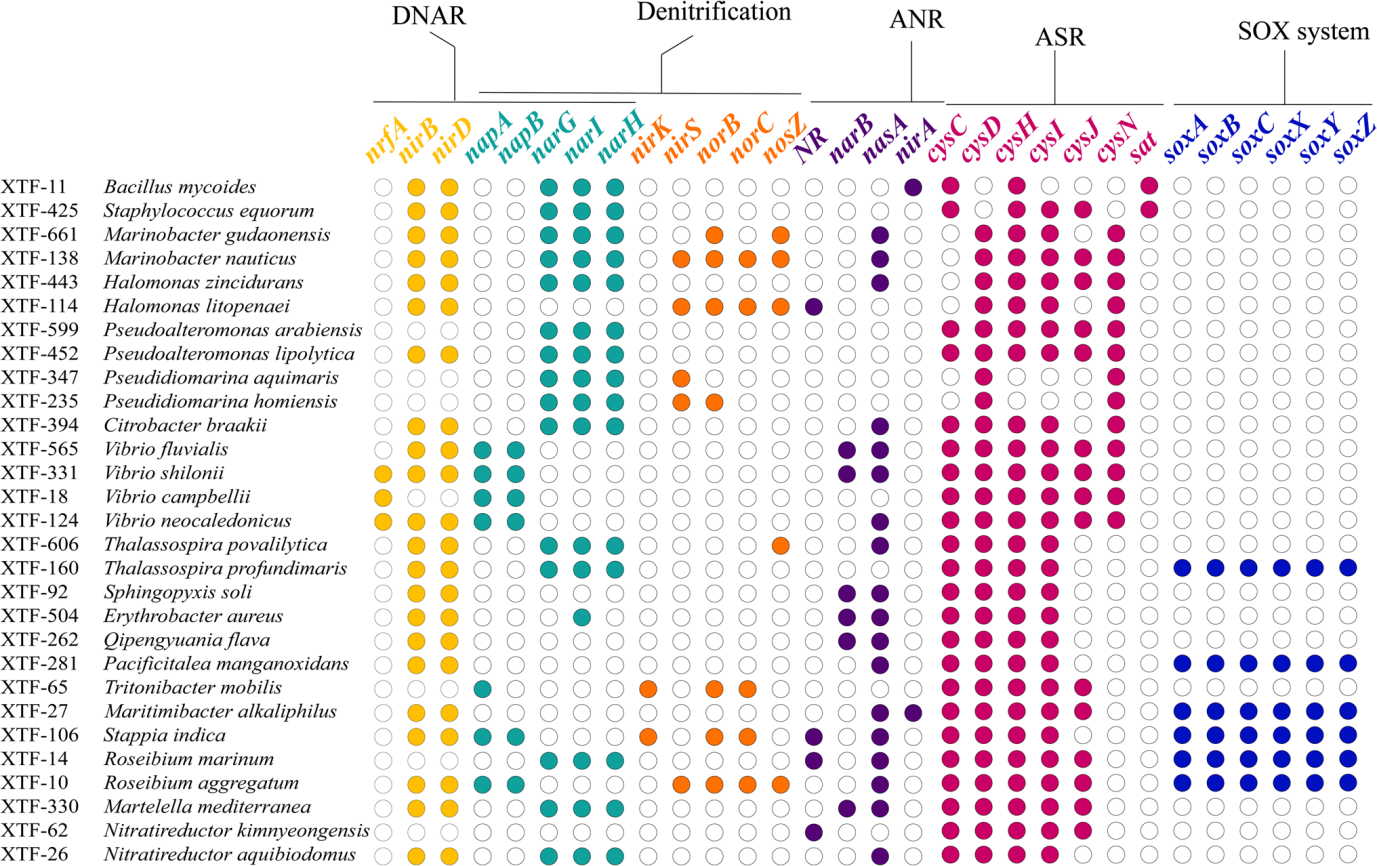
Corresponding genes of 29 strains representing different species involved in nitrogen and sulfur metabolism. Colored dots indicate presence while empty dots indicate absence of the genes in the different strains

Among 295 nitrate-reducing bacteria, 29 strains representing different species were selected for further metabolic study (Fig. S5). Here, we annotated the genomes of the 29 nitrate-reducing isolates, with the goal of further understanding their metabolic potentials based on the presence or absence of key genes for the pathways of nitrogen metabolism (Fig. 2). Whole-genome analysis indicated that all 29 nitrate-reducing isolates possessed genes responsible for dissimilatory nitrate reduction or ANR, which is consistent with their ability to reduce nitrate to nitrite. Of the 29 nitrate-reducing isolates, 62% (18/29) and 3% (1/29) contained genes for reducing nitrate to ammonia by DNRA (*napA*, *napB*, *narGHI*, *nirB*, *nirD*, *nrfA*) and ANR (*nasA*, *NR*, *narB*, *nirA*), respectively. Nitrate can be retained as ammonium via DNRA or as organic nitrogen via ANR. Both processes can contribute to maintaining a balance in the global nitrogen cycle by reducing the loss of nitrogen from ecosystems [62]. For example, DNRA played a significant role in conserving nitrogen in paddy soils, contributing up to 18% of the total nitrogen conservation [63]. In the photic zone of WNPO, inorganic N nutrients are usually insufficient to support microbial growth. Thus, these DNRA and ANR bacteria which represent a nitrogen-retaining pattern can be important for of biogeochemical cylce nitrogen. It has been reported that DNRA can be coupled with anammox by providing ammonium [64]. Our study indicated that DNRA was probably involved in conversion of nitrate to ammonia for nitrate-reducing bacteria and might be also a potential way to supply ammonium for non-nitrate-reducing microbes and ammonia-oxidizing microbes in euphotic zone of oligotrophic open ocean.

Interestingly, our genome sequence analysis revealed that *Marinobacter nauticus* XTF-138, *Halomonas litopenaei* XTF-114, and *R*. *aggregatum* XTF-10 possessed genes involved in the final three steps of the denitrification pathway (*nirS*, *norBC*, and *nosZ*), and thus may function in a manner beyond ANR or DNRA. In our nitrate reduction tests, the successive addition of Griess reagents and zinc dust to the nitrate culture medium followed by incubation at 25°C for 5 days did not lead to any color development by these three strains, indicating that nitrate was completely reduced (Fig. S5). Based on the genome sequence data and the ability of these strains to completely reduce nitrate, we speculate that they may use denitrification to reduce nitrate to molecular nitrogen via nitrite, nitric oxide (NO), and nitrous oxide (N_2_O).

### Sulfur Metabolism of Nitrate-Reducing Strains

During the assimilatory sulfate reduction (ASR) pathway, sulfate is initially activated to generate adenosine monophosphate (AMP), which is then converted to 3′-phosphoadenosine-5′-phosphosulfate sulfite (PAPS) and sulfide through a series of enzymatic steps involving *sat*, *cys*ND, *cys*C, *cyc*H, *cys*JI, and other enzymes [65]. All of the nitrate-reducing isolates identified herein contained genes involved in the ASR pathway (Fig. 2), which is utilized to synthesize sulfur-containing amino acids from sulfate [65]. Bacterial denitrification and sulfate reduction can often coexist in natural water systems [66]. Given that there were also heterotrophic denitrifiers, we speculate that denitrification and sulfate reduction processes may co-occur in the seawater of the photic zone.

Four proteins in the periplasm, encoded by *sox*YZ, *sox*XA, *sox*B, and *sox*CD, are essential for the sulfur oxidization (SOX) pathway [67]. Interestingly, we detected six nitrate-reducing isolates (*Thalassospira profundimaris* XTF-160, *Pacificitalea manganoxidans* XTF-281, *Roseibium marinum* XTF-14, *Maritimibacter alkaliphilus* XTF-27, *S. indica* XTF-106, and *R*. *aggregatum* XTF-10) that encoded SOX genes (*sox*XA, *sox*B, *sox*C, *sox*YZ). To validate their potential ability to oxidize thiosulfate, we incubated these six strains in modified DSMZ Medium142 liquid medium at 25°C for 3–5 days. The liquid medium turned from red to yellow, indicating that all of the tested strains effectively oxidize thiosulfate (Fig. S6). The thiosulfate oxidation which provides energy facilitates nitrate or nitrite reduction [68]. Together, the results showed that nitrate-reducing thiosulfate-oxidizing bacteria exist in the photic zone of the Western North Pacific Ocean.

DMSP is ubiquitous in the euphotic layers of the marine system, with a wide variation in concentrations ranging from nanomolar to micromolar [69]. DMSP, which is released into the environment through lysis, provides a substantial source of carbon and reduced sulfur for heterotrophic bacterial communities [48]. Among the isolates, we found that *T*. *profundimaris* XTF-160 and *Tritonibacter mobilis* XTF-65 contained a methionine methyltransferase gene (*mmt*N), which is a marker for bacterial synthesis of DMSP via the methionine methylation pathway [70] (Table S4). *R*. *marinum* XTF-14, *R*. *aggregatum* XTF-10, and *M*. *alkaliphilus* XTF-27 contained the DMSP synthesis gene, *dsy*B, which encodes the key methyltransferase enzyme and is a reliable reporter for bacterial DMSP synthesis in marine *Alphaproteobacteria* [71]. Therefore, these five nitrate-reducing isolates each contained a DMSP synthesis gene (*dsy*B or *mmt*N) in their available genomes.

We also found some nitrate-reducing isolates that may be involved in the DMSP cleavage pathway. For example, *H*. *litopenaei* XTF-114 contained the gene *ddd*D, *T*. *mobilis* XTF-65 contained the gene *ddd*P, and *R*. *marinum* XTF-14 and *R*. *aggregatum* XTF-10 contained the gene *ddd*L. These three genes mediate the cleavage of DMSP to dimethylsulfide (DMS), and thus are important for the ocean-atmosphere sulfur flux [72]. In the photic zone of the oligotrophic ocean, DMSP concentrations in seawater vary from 1 to 100 nM, and are normally highest in the Chl-*a* maximum layers [48]. Of the six isolates found to be involved in the synthesis and/or cleavage of DMSP, we found that they located near the Chl-*a* maximum layer (Table S5) and may participate in synthesizing and catabolizing DMSP in photic seawater.

### Organic Carbon Metabolism of Nitrate-Reducing Strains

To identify the potential for nitrate-reducing bacterial degradation and metabolism of complex carbohydrates in the water column of the photic zone, we performed functional annotation of the identified genes by comparison to the carbohydrate-active enzymes (CAZymes) database. A total of 2197 genes belonging to five CAZyme superfamilies were identified from the genomes of the 29 nitrate-reducing strains; of them, 40%, 36%, 5%, 3.8%, 1.7%, and 12.8% corresponded to GT (glycosyltransferase), GH (glycoside hydrolase), CE (carbohydrate esterase), AA (auxiliary activity), PL (polysaccharide lyase), and CBMs (carbohydrate-binding modules), respectively (Fig. S7A; Table S6).

Six classes of enzymes for complex polysaccharide degradation were predicted from the genomes (Fig. S7B; Table S6). The highest number of genes was found in the class of chitin degradation, for which family GH23 showed the highest abundance, followed by CBM5 (chitin-binding), and GH18 (chitinase). GH is often found with a CBM, which facilitates the efficient binding of the enzyme to carbohydrates [73]. Other classes of CAZymes in this study were associated with the degradation of lichenin, cellulose, pectin, starch, and trehalose. Chitin is widely found in fungi, and certain viruses of the photic zone in the marine environment, and chitinases possess the extraordinary ability to hydrolyze highly insoluble chitin polymer directly to lower molecular weight chitooligomers [74, 75]. Chitin is the most abundant aminopolysaccharide and interacts with both carbon and nitrogen cycles in the oceans, and most of the chitin found globally is produced near the surface of the aquatic environment [76]. The presence of multiple genes for chitin degradation may enable most of the nitrate-reducing isolates to use chitin as an important source of carbon and nitrogen, and thereby survive and reproduce in an oligotrophic condition.

In this work, 563 putatively secreted peptidases were identified and assigned to 20 families; of them, 60.8%, 29.2%, 5.7%, 3.9%, and 3.6% belonged to the metallo, serine, cysteine, threonine, and aspartic peptidase families, respectively (Fig. S7C; Table S7). Among these secreted peptidases, the metallo peptidase M38 represented the most abundant peptidase. Given proteinaceous compounds are important nitrogen nutrients for microorganisms and are deficient in the photic zone of WNPO, the extracellular peptidases of nitrate-reducing isolates are likely to play crucial roles in degrading organic nitrogen and thereby enabling the utilization of precious nitrogen sources.

## Conclusions

In the present research, 295/634 nitrate-utilizing isolates collected from the photic zone of the Western North Pacific Ocean could reduce nitrate to nitrite. Among these nitrate-reducing bacteria, *Q*. *flava*, *R*. *aggregatum*, *E. aureus*, *V. campbellii*, and *S. indica* were highly abundant in all seawater layers and found at almost all stations. Whole-genome analysis indicated that, consistent with the results of our nitrate-reduction tests, all 29 nitrate-reducing isolates possessed genes that could sustain dissimilatory nitrate reduction (three also possessed genes involved in the final three steps of the denitrification pathway) or ANR. All of the nitrate-reducing isolates contained genes involved in the ASR pathway and six also encoded SOX genes and had the ability to oxidize thiosulfate. We found that some nitrate-reducing isolates sampled from around the Chl-*a* maximum layer contained DMSP biosynthesis genes (*mmtN* and *dsyB*) and/or DMSP cleavage genes (*dddD*, *dddP*, and *dddL*), suggesting that they may be involved in DMSP metabolism in the seawater. Interestingly, the widely distributed *R. aggregatum*, which was a dominant nitrate reducer in our samples, possessed genes involved in the pathways of denitrification, sulfur oxidation, and DMSP biosynthesis and cleavage, prompting us to speculate that *R. aggregatum* may be a major mediator of nitrogen and sulfur metabolism in the photic zone. All of these nitrate-reducing strains were not involved in photosynthesis and methane metabolism. The presence of multiple genes for chitin degradation may be crucial for the survival of most nitrate-reducing isolates, as chitin may be an important carbon nutrient in these nutrient-poor ocean environments. Collectively, the results of this study provide important insights into the nitrogen, sulfur, and carbon biogeochemical cycles of nitrate-reducing strains in the oligotrophic marine photic zone of WNPO.

### Supplementary Information

ESM 1

ESM 2

High resolution image (TIF 110712 kb)

ESM 3

High resolution image (TIF 37470 kb)

ESM 4

High resolution image (TIF 22815 kb)

ESM 5

ESM 6

ESM 7

ESM 8

ESM 9

ESM 10

ESM 11

ESM 12

ESM 13

ESM 14
