# Supplementary figures and images for: The Diversity and Metabolism of Culturable Nitrate-Reducing Bacteria from the Photic Zone of the Western North Pacific Ocean
Source: Microb Ecol. 2023 Aug 8;86(4):2781–9. doi: 10.1007/s00248-023-02284-w (PMC10640468; doi:10.1007/s00248-023-02284-w)

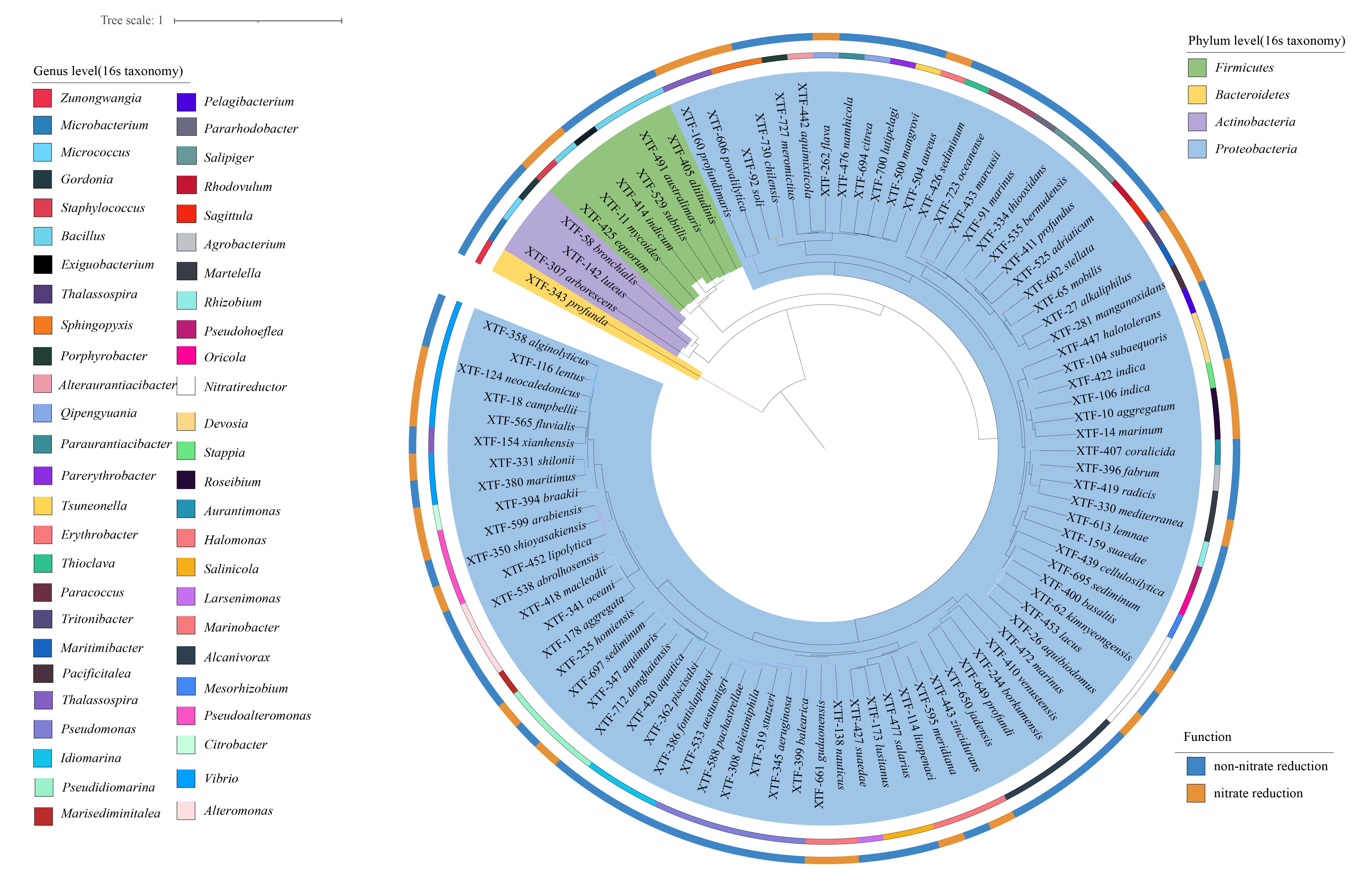

Supplement: Supplementary file 1 — ESM 1 [file 248_2023_2284_MOESM1_ESM.jpg]

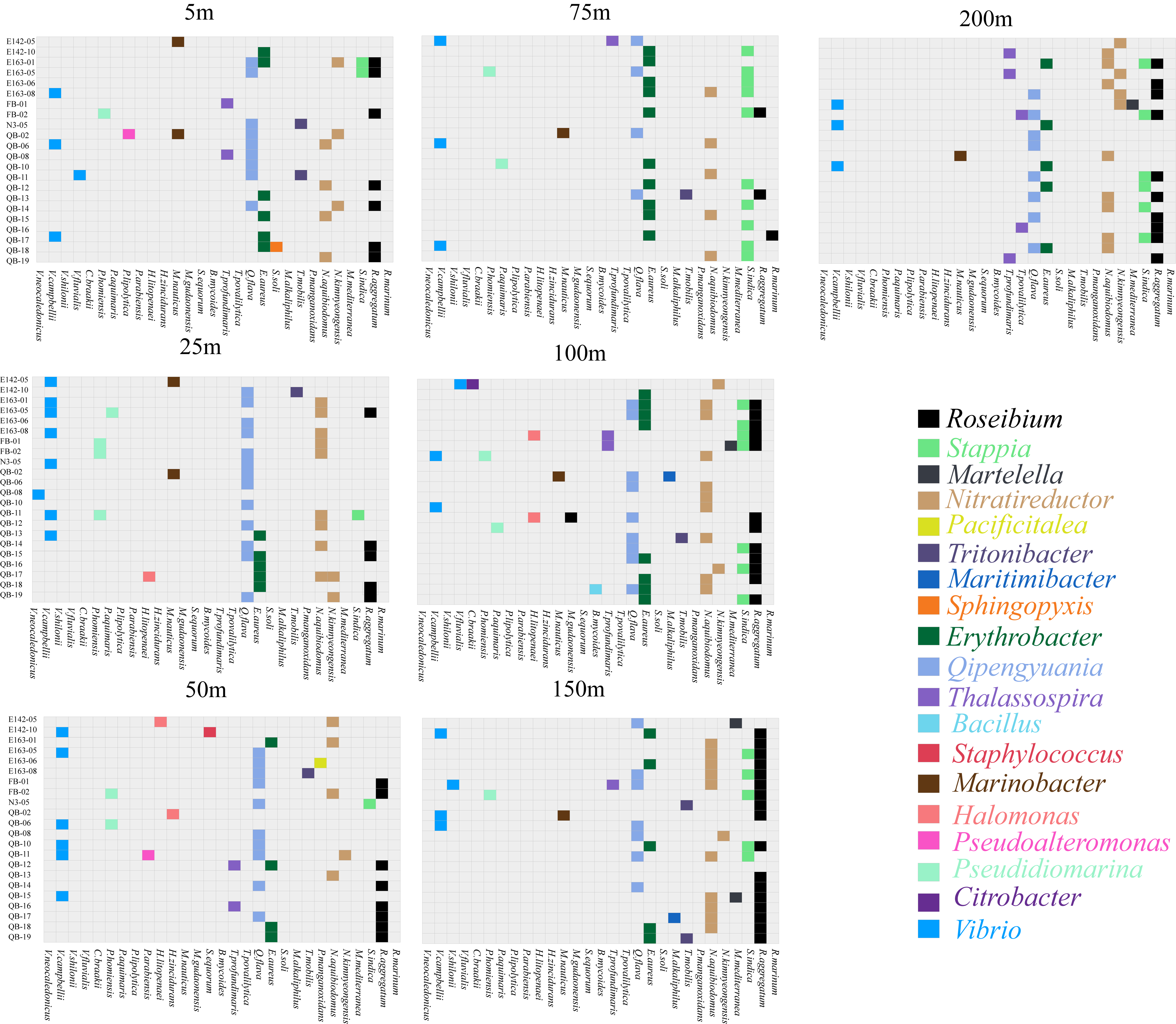

Supplement: Supplementary file 2 — ESM 2 [file 248_2023_2284_Fig15_ESM.png]

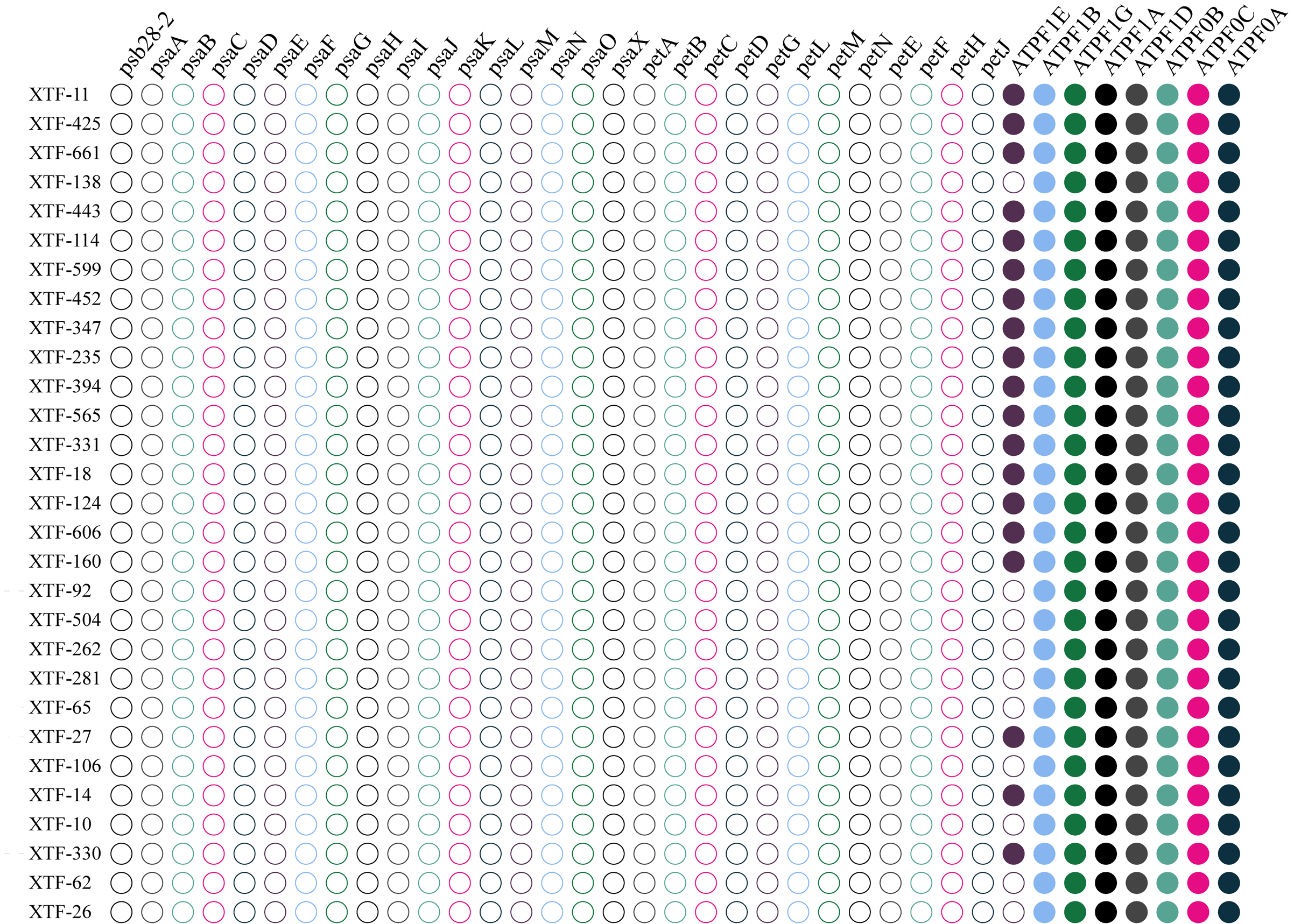

Supplement: Supplementary file 4 — ESM 3 [file 248_2023_2284_Fig16_ESM.png]

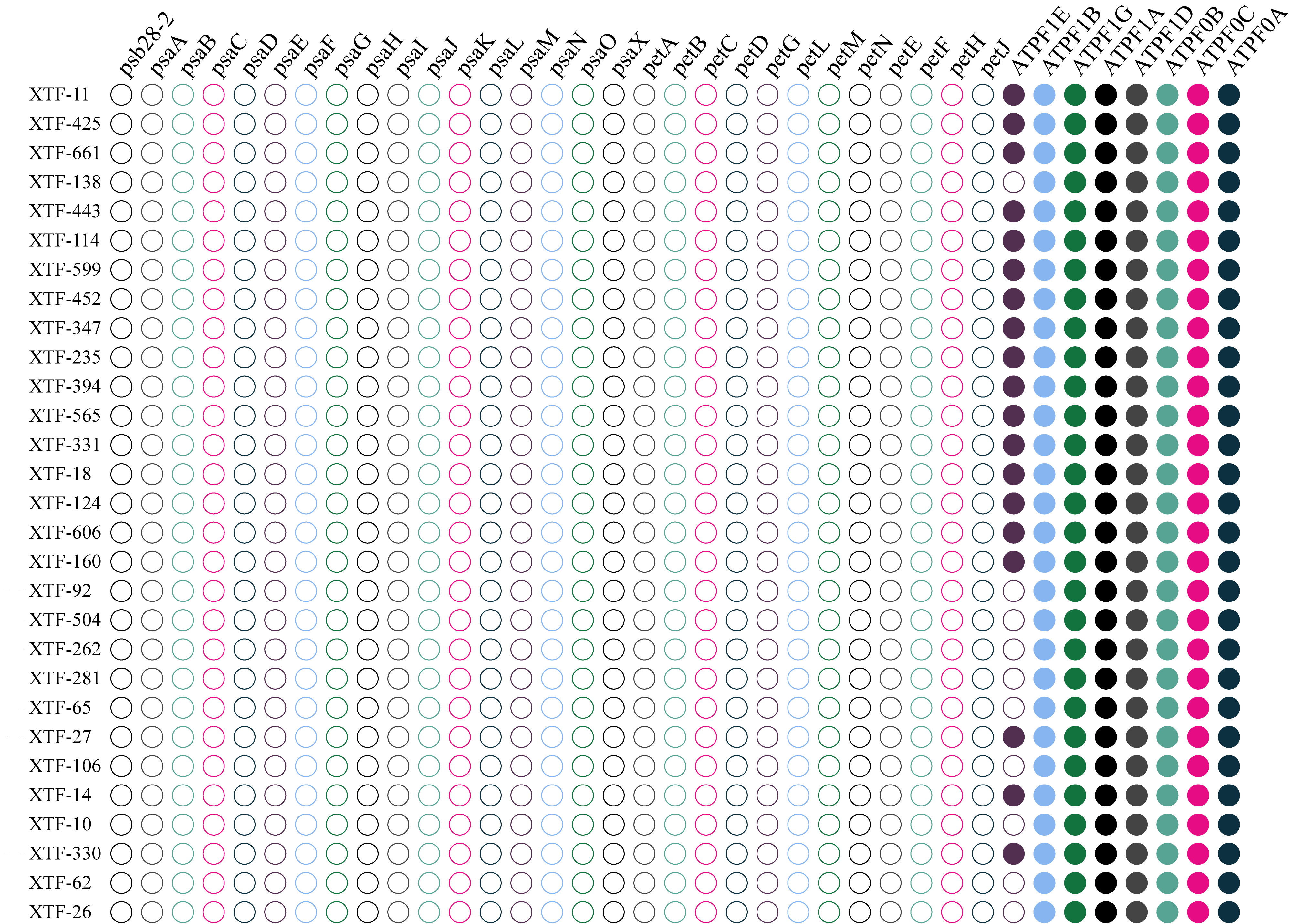

Supplement: Supplementary file 5 — High resolution image (TIF 37470 kb) [file 248_2023_2284_MOESM3_ESM.tif]

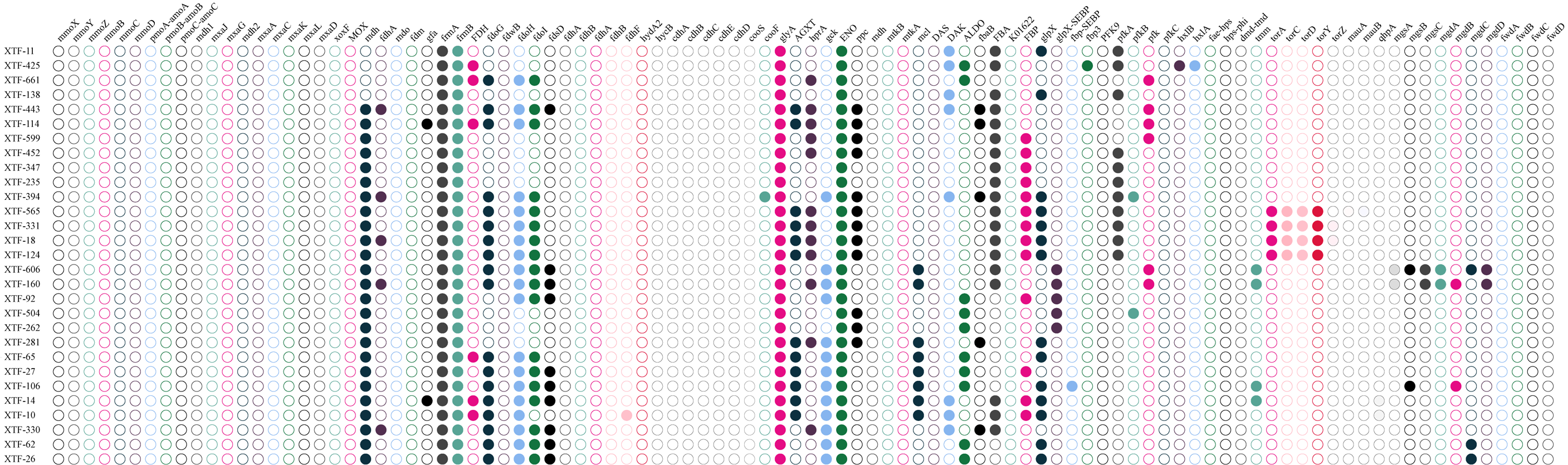

Supplement: Supplementary file 6 — ESM 4 [file 248_2023_2284_Fig17_ESM.png]

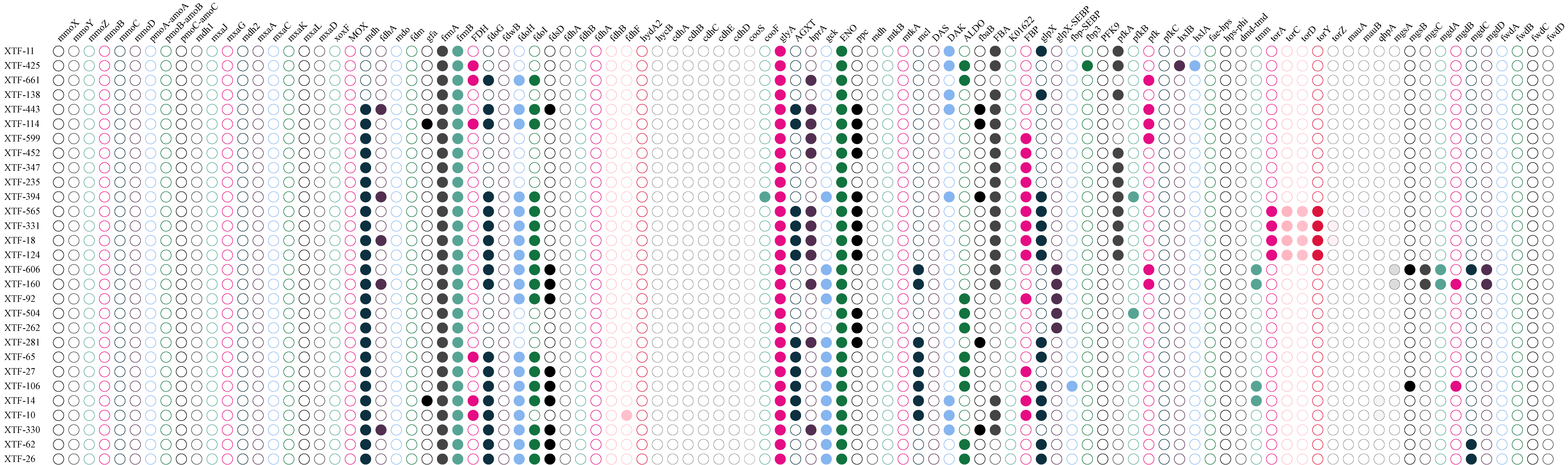

Supplement: Supplementary file 7 — High resolution image (TIF 22815 kb) [file 248_2023_2284_MOESM4_ESM.tif]

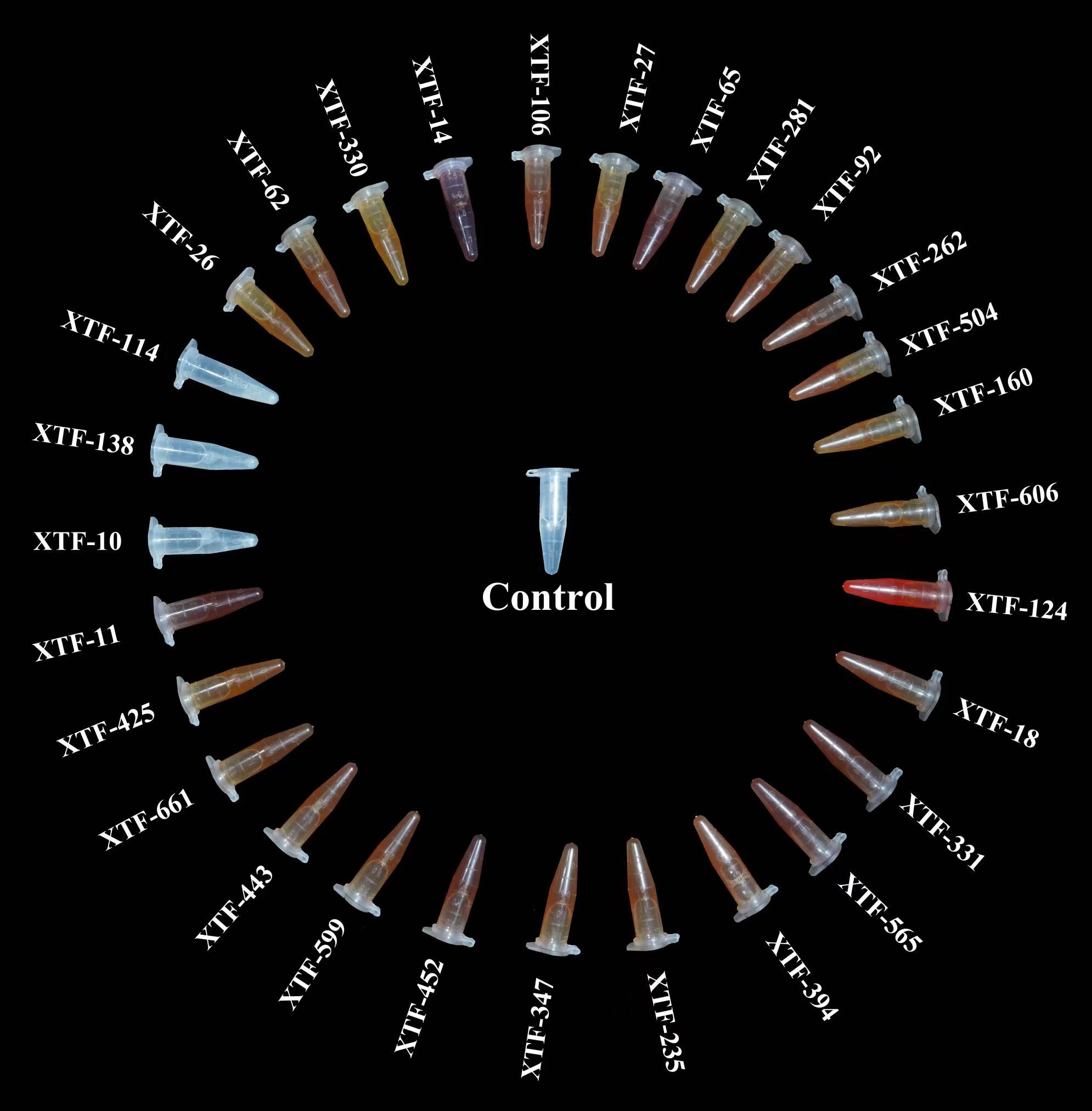

Supplement: Supplementary file 8 — ESM 5 [file 248_2023_2284_MOESM5_ESM.jpg]

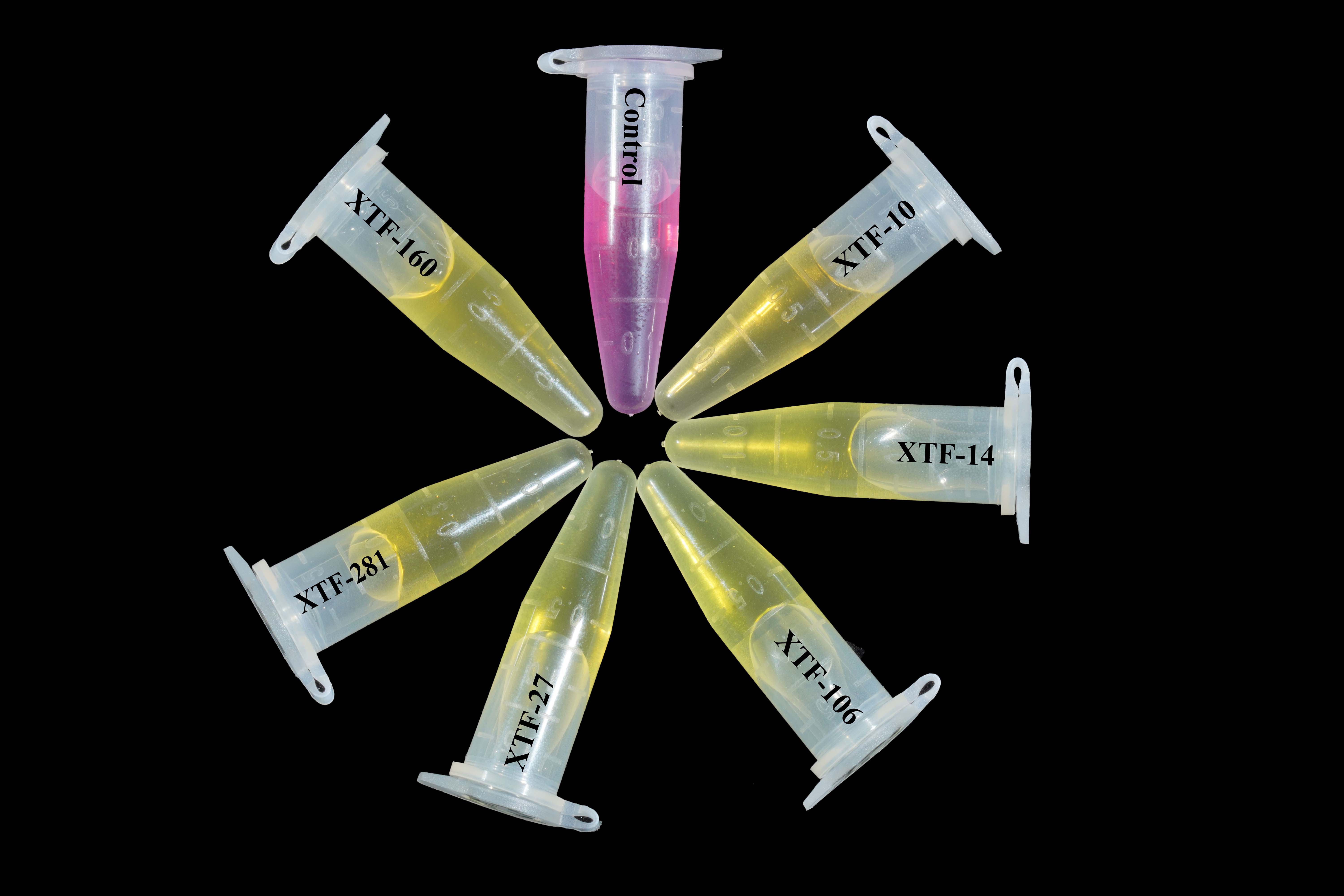

Supplement: Supplementary file 17 — ESM 14 [file 248_2023_2284_MOESM14_ESM.jpg]
